# Letter to the Editor: There Should Be a Clear Distinction Between Legitimate Protest and Antisemitism

**DOI:** 10.5041/RMMJ.10546

**Published:** 2025-04-29

**Authors:** Yasmeen Abu Fraiha, Akiva Leibowitz

**Affiliations:** 1 Department of Anesthesia and Critical Care, Beth Israel Deaconess Medical Center, Boston, MA, USA; 2 Middle East Initiative, Belfer Center for Science and International Affairs, Harvard Kennedy School, Cambridge, MA, USA

**Keywords:** Activism, antisemitism, bias, Israel-Gaza war, protest

**Dear Editor**,

The recently published paper “US Medical Schools’ 2024 Commencements and Antisemitism: Addressing Unprofessional Behavior” discusses antisemitic expressions and unprofessional behavior in US medical schools’ 2024 commencement ceremonies.^1^ While we share the authors’ concerns regarding rising antisemitic, anti-Palestinian, and anti-Muslim bias, alongside hateful behavior toward minorities and immigrants in the US in general and in medical schools in particular, we are also concerned about the significant bias informing this paper. The authors mistakenly conflate antisemitism with harsh criticism of the Israeli government and the actions of its military as well as legitimate acts of solidarity with people under oppression. This fallacy is further aggravated by serious concerns (mentioned by the authors themselves) involving the paper’s methodological and statistical shortcomings. Ultimately, the paper lacks scientific rigor and appears to be ideologically motivated rather than a contribution to objective research.

A major flaw in this manuscript is the presentation and perception of Palestinian cultural symbols such as the Palestinian flag, the keffiyeh, Al Aqsa Mosque, and the map of Palestine as antisemitic. A generous reading would suggest that this conflation is based on deep-seated ignorance, while a less generous reading raises concerns of an effort to maliciously manipulate readers who may not be attuned to the cultural and historical value of these symbols. The keffiyeh is a cultural cloth that has been worn for centuries by Arab Bedouin farmers to protect them from the strong Middle Eastern sun, and it has multiple variations including the famous red-and-white keffiyeh and the more delicate fully-white one worn with the black agal (a head cord).^2^ In fact, the native Jews of Historic Palestine/Eretz Yisrael, immigrants from Arab countries (“Mizrahi” Jews), and Zionists members of HaShomer (a Jewish defense organization in Palestine established by social Zionists in 1909) and other paramilitary groups often assumed the local dress code which included the traditional head coverings, the keffiyeh amongst them.^3^ To be sure, under the oppression of both the Ottoman and the British Empires, the keffiyeh became a symbol of the Arab struggle for liberation. In the 1960s, the leader of the Palestinian Liberation Organization—Yasser Arafat—adopted the black-and-white keffiyeh as a symbol of the Palestinian struggle for freedom and independence. For many, donning the keffiyeh today conveys a strong sense of cultural belonging and heritage while also symbolizing solidarity with the Palestinian struggle for self-determination and freedom, as well as solidarity with oppressed people worldwide.^4^

The keffiyeh is only one example of how the authors assert their own biased perspectives and interpretations to symbols, rather than basing it on firm historical and cultural evidence. The examples purposed as “antisemitism” in the manuscript, including the Palestinian flag, the “empty” map of Israel/Palestine, the signs stating “occupation is health crisis” and “stop bombing hospitals,” or the chant “from the river to the sea” are not antisemitic tropes but rather forms of commitment to different aspects of social justice, which are recognized determinants of health.^5^,^6^ Even by the standard set forward by the controversial International Holocaust Remembrance Alliance (IHRA) working definition of antisemitism, these symbols do not amount to antisemitism. Critical perspectives on the IHRA definition, such as the Jerusalem Declaration on Antisemitism, endorsed by 300 scholars across the globe, including Derek Penslar, Amos Goldberg, Alon Confino, Stefanie Schüler-Springorum, Omer Bartov, Susannah Heschel, and Abigail Jacobson, explicitly exclude such expressions in this context from being considered antisemitic. While these symbols might cause discomfort to some, they do not, as the authors claim, “call for aiding or justifying the killing or harming of Jews in the name of a radical ideology or extremist view of religion, or denying the Jewish people their right to self-determination.” The authors do not even attempt to demonstrate that these symbols are manifestations of antisemitism and, therefore, fail to meet the most fundamental scientific requirements, hence undermining the very foundations of their article.

Over the past year, there has been an active debate regarding the claim backed by organizations such as the Anti-Defamation League that pro-Palestinian chants, symbols, and demonstrations (many of which were co-attended by Jewish groups) are examples of antisemitic, inflammatory, and hateful speech. Scholars worldwide, Jews and Israelis amongst them, have demonstrated that these are legitimate protests, and the interpretation of their messaging as antisemitic is just another way to silence Palestinian voices calling for freedom and liberation, and delegitimizing critique on the rights-abusive policies and war crimes of the Israeli government.^7^ The Nexus Project, led by a task force of Jewish scholars and leaders with the aim to combat true antisemitism and protect free speech, recently published a campus guide that looks into the complexity of terms such as “apartheid,” “anti-Zionism,” “genocide,” and others, stating in which exact circumstances they are examples of antisemitism, with the understanding that this is not necessarily always or even predominantly the case. However, the authors of “US Medical Schools’ 2024 Commencements and Antisemitism” neglect to mention such studies or the complexities they expose in the discussion. Even the attempt to argue that these identity symbols and acts of solidarity constitute “unprofessional behavior” is not supported by the evidence, with the authors themselves admitting that, except for two medical schools, universities do not have clear rules against donning such symbols or expressing solidarity.

Reality is perceived by humans through the prism of values they hold, leading to a wide variety of interpretations of any event.^8^ However, researchers should be aware of their own biases and differentiate opinion and belief from factually based scientific discovery. A scholar interested in understanding why students donned a keffiyeh during commencement would interview those who wore them to ask what their intentions were, would investigate the history of the keffiyeh and its different uses over time, and would compare the donning of this cultural artifact with other cultural artifacts donned in different contexts. We educate our trainees to consider all available empirical evidence, to be sensitive to patients’ cultural backgrounds, and to be attuned to what they might perceive as injustices affecting health and disease.^9^ Indeed, speaking up, verbally or symbolically, may cause discomfort to colleagues, but commitment to the wellbeing of our patients—personal or collective, those lying in front of us or those many miles away—remains a priority. Physicians engaged in their surrounding society cannot remain silent amidst horrific human-made humanitarian catastrophes, including the one that has been unfolding in Gaza over the past 15 months.^10^ Deeply embedded in Jewish tradition is the obligation to stand up to injustice which is costing lives—“You shall not go around as a gossipmonger amidst your people. You shall not stand by [the shedding of] your fellow’s blood. I am the Lord” (Leviticus 19:16).

Finally, quoting Moses Maimonides, we should keep in mind his words from *The Guide for the Perplexed*:

We naturally like what we have been accustomed to, and are attracted towards it. […] The same is the case with those opinions of man to which he has been accustomed from his youth; he likes them, defends them, and shuns the opposite views. This is likewise one of the causes which prevent men from finding truth, and which make them cling to their habitual opinions.^11^

## Abbreviations

IHRA: International Holocaust Remembrance Alliance
